# Adhesive Capsulitis Secondary to COVID-19 Vaccination - A Case Series

**DOI:** 10.5704/MOJ.2307.007

**Published:** 2023-07

**Authors:** BCM Foong, SWL Ho, LTJ Tan, KT Lee, T Jegathesan

**Affiliations:** Department of Orthopaedic Surgery, Tan Tock Seng Hospital, Singapore

**Keywords:** shoulder joint, adhesive capsulitis, COVID-19 vaccines

## Abstract

**Introduction:**

Shoulder injury related to vaccine administration (SIRVA) is a group of pathologies defined by pain and stiffness after intramuscular administration of vaccine to the upper arm and has been reported after COVID-19 vaccination. We aim to discuss its pathophysiology, clinical presentation, treatment and outcomes.

**Materials and methods:**

We retrospectively identified patients presenting with adhesive capsulitis within four weeks of administration of COVID-19 vaccine to the affected arm at our tertiary institution from March 2021 to December 2022.

**Result:**

Based on the above criteria, we identified seven cases of adhesive capsulitis, comprising one male and six female patients, with average age of 60 years. We present initial symptoms, signs and the duration from when the vaccine was administered. We have highlighted our treatment strategies as well as the clinical and functional outcomes reported by these patients after treatment. We have reported improvement in both Visual Analogue Scale (VAS) and range of motion (ROM) in all our patients after non-surgical management which included physiotherapy and, in some cases, hydrodilatation.

**Conclusion:**

SIRVA related adhesive capsulitis is rare and under-reported with limited information in current literature. This study highlights that adhesive capsulitis is a potential complication arising from improper COVID-19 vaccine administration and reinforces traditional wisdom of administering vaccinations on the non-dominant arm. Conservative treatment strategies appear to be effective, particularly hydrodilatation combined with physiotherapy, and patients are expected to have a good return of function.

## Introduction

Coronavirus disease 19 (COVID-19) pandemic was first detected in late 2019 but has since spread globally, infecting more than 240 million people worldwide and causing more than 5 million deaths^1^. Many countries around the world have been undertaking large scale campaigns to vaccinate their populations against the virus. Over 7 billion doses have been administered globally with an estimate of 45.7% of the global population being fully vaccinated^2^ in 2022.

COVID-19 vaccines are not without side effects. Examples of systemic side effects include fever, headaches, fatigue, myalgia and arthralgia. Examples of localised side effects include pain, swelling and redness at the injection site with adjacent lymphadenitis^3^. A wide range of vaccines have been previously associated with shoulder injury related to vaccine administration (SIRVA), the influenza vaccine being the most common. More recently, COVID-19 vaccines have also been reported to cause SIRVA4. SIRVA includes a group of shoulder pathologies that are defined by the development of shoulder pain, stiffness and sometimes weakness after administration of intramuscular vaccination to the deltoid muscle^5-^9. These include adhesive capsulitis, localised bursitis and tendinitis^5-8^. Adhesive capsulitis is characterised by a significant restriction of both active and passive shoulder motion that occurs in the absence of a known intrinsic shoulder pathology^10^.

SIRVA is thought to be triggered by the improper injection of intramuscular deltoid vaccines such as incorrect location, direction or depth of needle penetration. It is proposed that the vaccine is injected into structures deep to the deltoid muscle including the subdeltoid bursa or even the shoulder joint, setting off a local inflammatory response^5,7,8^. This can lead to surrounding bursitis, rotator cuff tendinitis and adhesive capsulitis^5-8^.

The purpose of this study is to report a series of patients who developed adhesive capsulitis after COVID-19 vaccination with a focus on clinical presentation, treatment and outcomes. We will also discuss the pathophysiology of this rare but possible outcome following COVID vaccine administration.

## Materials and Methods

We identified all patients having treatment for adhesive capsulitis at our tertiary institution from March 2021 to December 2021. Clinically, patients were diagnosed with adhesive capsulitis based on a complete medical history and thorough physical examination performed by experienced orthopaedic surgeons. Diagnosis involved a restriction of both active and passive forward elevation and external rotation in the absence of an intrinsic shoulder disorder. Patients who had the onset of symptoms after administration of COVID-19 vaccine to the affected arm were included. All patients who presented with probable secondary causes of frozen shoulder following the vaccine were excluded, and these included patients with recent trauma or prolonged immobilization from stroke disease.

This study was approved by the National Healthcare Group Domain Specific Review Board and was conducted in conformity with ethical principles of research.

Data was collected retrospectively. Vaccine data was collected and included the vaccine manufacturer and date of administration. Patients were assess based on Visual Analogue Scale (VAS) and range of motion (ROM). This data was collected during the first consultation prior to initiation of treatment and during the final consultation prior to the patient’s discharge. Patients had an initial consultation and was then reviewed 1 and 6 months after their initial presentation. We employed several modes of treatment. Analgesia was standardised to include paracetamol as well as oral and topical non-steroidal anti-inflammatory drugs (NSAID). Physiotherapy included fortnightly sessions which involved a standard regimen of exercises focusing on stretching and holding the shoulder joint in flexion, abduction, external rotation and internal rotation.

Hydrodilatation involved introduction of a spinal needle into the glenohumeral joint under fluoroscopic guidance. Iohexol contrast media was diluted in normal saline and injected into the joint until contrast extravasation was noted. A mixture of triamcinolone and bupivacaine was then injected into the joint.

## Results

We identified seven cases of adhesive capsulitis occurring within four weeks after administration of COVID-19 vaccine to the affected arm. They comprised of one male and six female patients with an average age of 60 years. Table I summarises the patient demographics, vaccine information, clinical presentation, treatment as well as outcomes after treatment.

**Table I:** Summary of patient demographics, clinical presentation, treatment and outcomes

Patient	Age	Co-morbidities	Affected Shoulder (Vaccinated Arm)	Type of Vaccine (Dose Implicated)	Onset of symptoms	Post vaccination ROM	Post vaccination VAS	Treatment	Duration of Treatment	Post treatment ROM	Post treatment VAS	US findings
1	51	Nil	Left (left)	Moderna (2)	3 weeks	FF-120 ER-30	8/10	PT and HD	HD-4 weeks PT-3 weeks	FF-140 ER-70 IR-T7	6/10	Nil
2	55	T2DM IHD	Left (Left)	Modern (2)	1 week	FF-150 ER-70 IR-T12	10/10	PT and HD	HD-4 weeks	FF-160 ER-70 IR-T4	2/10	Supraspinatus and subscapularis tendinosis. SASD bursitis
3	75	Hypertension, hyperlipidaemia	Left (Left)	Moderna (2)	4 weeks	FF-110 ER-10 IR-Sacrum	8/10	PT and HD	HD-2 weeks PT-4 weeks	FF-140 ER-50 IR-L1	4/10	Supraspinatus and subscapularis tendinosis. SASD bursitis
4	48	Nil	Left (Left)	Moderna (2)	4 weeks	FF-90 ER-20 IR-L3	6/10	PT and HD	HD-6 weeks PT-4 weeks	FF-140 ER-20 IR-L1	2/10	Nil
5	59	Nil	Left (Left)	Pfizer (2)	1 week	FF-100 ER-20 IR-Sacrum	10/10	PT and HD	HD-6 weeks PT-5 weeks	FF-140 ER-30 IR-L1	5/10	Nil
6	71	T2DM hyperlipidaemia	Left (Left)	Pfizer (2)	1 day	FF-120 ER-40 IR-L3	7/10	PT	6 weeks	FF-120 IR-Back	1/10	Nil
7	66	Nil	Left (Left)	Pfizer (2)	3 weeks	FF-140 ER-40 IR-Sacrum	8/10	PT	8 weeks	FF-160 ER-60 IR-L1	3/10	Supraspinatus and subscapularis tendinosis.

Abbreviations - VAS: Visual Analogue Scale for pain, FF: Forward Flexion, ER: External Rotation, IR: Internal Rotation, SASD: Subacromial subdeltoid, T2DM: Type 2 Diabetes Mellitus, IHD: Ischaemic Heart Disease, PT: Physiotherapy, HD: Hydrodilatation

Our patients presented with shoulder pain and stiffness between one day and 4 weeks after receiving the either the first or second dose of the COVID-19 vaccine, with an average of 14.1 days duration. Patients who developed symptoms after the second dose were asymptomatic after the first dose. All the patients received the vaccine on their left arm. Vaccines were administered by registered nursing staff. Four patients (57.1%) received the Moderna mRNA-1273 and 3 patients (42.9%) received the Pfizer-BioNTech BNT162b2 vaccine.

They presented with an average VAS of 8.1 and globally reduced ROM. The mean ROM include forward flexion of 104.3° and external rotation of 32.3°. Internal rotation ranged between the level of the sacrum and T7. All patients were investigated with anteroposterior and lateral radiographs of the affected shoulder. Where the diagnosis was less certain, patients were further investigated with ultrasonography of the affected shoulder. Advanced imaging was not routinely performed for all patients. All our patients had normal radiographs and the findings on ultrasound are highlighted in Table I. All patients were prescribed with analgesia and underwent physiotherapy. Five of our patients (71%) underwent hydrodilatation of the affected shoulder joint. All patients were followed-up for one year from the time of presentation.

The mean post-treatment VAS score was 3.3. The mean post-treatment ROM was reported as forward flexion of 140° and external rotation of 48.6°. Improvement in pain and ROM was seen especially for patients who underwent both physiotherapy and hydrodilatation of the shoulder.

## Discussion

SIRVA has been described in the administration of several different vaccines including influenza, pneumococcal, diphtheria-tetanus-pertussis, diphtheria-tetanus toxoid, human papillomavirus, hepatitis A and more recently COVID-194,5,8. More emphasis tends to be placed on severe side effects of vaccines such as acute allergic reactions and less so on mild side effects such as shoulder pain, possibly resulting in under-reporting. As such, there is limited data on SIRVA in current literature and the exact prevalence is unknown^4^.

SIRVA secondary to COVID-19 vaccine has been reported in literature^8,11-13^. Our study shows that SIRVA related adhesive capsulitis and primary adhesive capsulitis present similarly with primary complaints of shoulder pain and stiffness. However, for the patients in our series, the onset of the pain was acute and within a relatively short period from administration of the COVID-19 vaccine to the ipsilateral shoulder. This differs from primary adhesive capsulitis which tends to have a more gradual onset and progression^14^. This could be attributed to the acute inflammation caused by the inappropriate injecting of vaccine material. We also demonstrate that the treatment principles for conventional idiopathic adhesive capsulitis can also be applied for SIRVA related adhesive capsulitis. Our patients were treated with a combination of physiotherapy and intra-articular hydrodilatation and demonstrated good improvement in both VAS scores as well as ROM.

Improper injection technique is a commonly thought to cause SIRVA. This includes incorrect site and angle of injection as well as overpenetration of the needle into subdeltoid tissue^4,5,7^. Bodor *et al* described the subacromial bursa to lie 3.0 to 6.0 cm inferior from the acromion and just 0.8 to 1.6 cm deep to the skin^7^. This makes it susceptible to being penetrated if vaccines are administered too proximally or too deeply. In several studies, patients with SIRVA have described the vaccine being administrated ‘too high’ in the deltoid muscle which potentially resulted in penetration of the subacromial bursa^4,5,7^. This can result in introduction of vaccine compounds into synovial tissue, triggering a local inflammatory response which manifests as a range of shoulder pathologies including local bursitis, tendinitis, adhesive capsulitis and rotator cuff tears which have been correlated in MRI studies^5,6,15-17^. Atanasoff *et al* carried out a study of 13 patients presenting with SIRVA. MRI studies when done showed findings of subdeltoid effusions, bursitis and tendinitis^5^.

Despite good accuracy of vaccine administration, pain and heaviness in the arm are known and common side effects of vaccine administration^3^. This may translate into prolonged disuse and immobility of the arm following vaccination. Hence, patients are at risk of developing adhesive capsulitis due to this immobilisation. A combination of underlying inflammation and immunological response may aggravate this pain and stiffness, further potentiating the development of adhesive capsulitis. This is supported by existing data that physiotherapy of the shoulder joint has been described to be useful in preventing development of adhesive capsulitis in the context of SIRVA^9^. The development of adhesive capsulitis after vaccination is likely influenced by many factors such as improper vaccination technique and underlying inflammatory response. Immobilisation of the affected arm can further predispose patients to developing adhesive capsulitis post-vaccination.

SIRVA have been commonly managed with analgesia, especially non-steroidal autoinflammatory drugs (NSAID), physiotherapy, and corticosteroid injections^18^. Non-surgical management of adhesive capsulitis include physiotherapy, oral analgesia, corticosteroid injections and hydrodilatation of the shoulder joint capsule. Surgical options include manipulation under anaesthesia and arthroscopic capsular release^19^. The first line of treatment for patients presenting in the early stages of frozen shoulder is physiotherapy. A Cochrane study showed that there was little evidence to support physiotherapy alone in the treatment of adhesive capsulitis^20^. As such, it is recommended to combine physiotherapy with other forms of treatment. Five out of seven of our patients were treated with a combination of physiotherapy and shoulder joint hydrodilatation to good effect with notable improvements in ROM as well as VAS scores.

Studies have proposed certain measures to reduce the risk of developing SIRVA. Anatomical landmarks should be clearly defined to ensure administration in the proper site. Avoidance of the superior one third of the deltoid muscle has been advocated. Some guidelines have proposed different anatomical landmarks including 2-3 finger breadths distal from the acromion, the midpoint between the acromion and deltoid tuberosity and the middle third of the deltoid muscle bulk^21^. Having both patient and provider seated during administration has also been proposed to avoid injecting too proximally^5^. Healthcare providers that are involved in administration of COVID-19 vaccines should be trained and made aware of these measures to reduce the risk of SIRVA.

This case series is limited by the small sample population with seven patients presented. It is effective as an initial pilot study, showing that SIRVA related adhesive capsulitis is a possible complication of COVID-19 vaccination. Further studies to better understand the incidence and long-term outcomes can be performed as more of the global population receives COVID-19 vaccines.

## Conclusion

We report cases of adhesive capsulitis after administration of COVID-19 vaccine and reinforces traditional wisdom of administering vaccinations on the non-dominant arm. It can occur with both the Pfizer-BioNTech BNT162b2 and Moderna mRNA-1273 vaccine, often after administration of the second dose. We have demonstrated that post COVID-19 vaccination adhesive capsulitis is highly treatable, and patients have a good return of function with non-surgical forms of treatment. Considering the global push for more people to be vaccinated against COVID-19, it is important for physicians and patients to be aware of the possibility of adhesive capsulitis as a potential complication of vaccine administration but also appreciate that it is a highly treatable condition.
